# Autophagic Upregulation Is Cytoprotective in Ischemia/Reperfusion-Injured Retina and Retinal Progenitor Cells

**DOI:** 10.3390/ijms22168446

**Published:** 2021-08-06

**Authors:** Larissa Ho Ching Tang, Frederic Khe Cheong Fung, Angela Ka Wai Lai, Ian Yat Hin Wong, Kendrick Co Shih, Amy Cheuk Yin Lo

**Affiliations:** Department of Ophthalmology, Li Ka Shing Faculty of Medicine, The University of Hong Kong, Hong Kong; lhctang@connect.hku.hk (L.H.C.T.); fredericfung@hotmail.com (F.K.C.F.); kawai_ang@yahoo.com.hk (A.K.W.L.); ianyhwong@gmail.com (I.Y.H.W.); kcshih@hku.hk (K.C.S.)

**Keywords:** macroautophagy, hypoxia, autophagy inhibitors, LC3, LAMP1, NH_4_Cl, CoCl_2_, R28 cells

## Abstract

The cytoprotective versus cytotoxic role of macroautophagy in ocular ischemia/reperfusion injuries remains controversial and its effects under hyperglycemia are unclear. We investigated the involvement of autophagy in in vitro and in vivo normoglycemic and hyperglycemic models of retinal ischemia/reperfusion injury. Retinal ischemia (2 h) and reperfusion (2 or 22 h) was induced in wild-type and type I diabetic Ins2^Akita/+^ mice using a middle cerebral artery occlusion model. R28 retinal precursor cells were subjected to CoCl_2_-induced hypoxia with or without autophagic inhibitor NH_4_Cl. Autophagic regulation during ischemia/reperfusion was assessed through immunohistochemical detection and Western blotting of microtubule-associated protein 1A/1B-light chain 3 (LC3) and lysosomal associated membrane protein 1 (LAMP1). Effect of autophagic inhibition on cell viability and morphology under hypoxic conditions was also evaluated. Upregulation of autophagic markers in the inner retinae was seen after two hours reperfusion, with tapering of the response following 22 h of reperfusion in vivo. LC3-II turnover assays confirmed an increase in autophagic flux in our hypoxic in vitro model. Pharmacological autophagic inhibition under hypoxic conditions decreased cell survival and induced structural changes not demonstrated with autophagic inhibition alone. Yet no statistically significant different autophagic responses in ischemia/reperfusion injuries were seen between the two glycemic states.

## 1. Introduction

Autophagy is a catabolic process by which eukaryotic cells eliminate and recycle cytoplasmic materials for cellular homeostasis. Macroautophagy (referred to thereafter as autophagy), one of the three types of autophagy, is a highly dynamic process where its modulation and role is often considered to be highly dependent on the pathological stage and juncture at which it is being studied. Basal levels are required for cellular homeostasis, yet uncontrolled autophagy activation can result in both cytoprotective or cytotoxic effects [1]. Retinal ischemia/reperfusion (I/R) events occur in various ocular conditions such as diabetic retinopathy, retinal artery occlusion, amaurosis fugax, and glaucoma. While apoptosis has traditionally been the most extensively studied form of cell death in retinal I/R injuries, there has been increasing interest in the divergent role of autophagy and its modulation as a potential therapeutic strategy. Various in vivo retinal I/R injury studies using the increase in intraocular pressure (IOP) model in rats have shown varying durations of initial autophagic activation [2,3,4,5], ranging from three to six hours [2,5], or continuous upregulation for up to seven days [4]. Russo et al., however, reported the decrease in autophagic marker microtubule-associated protein 1A/1B-light chain 3 (LC3) LC3-II expression and Beclin-1 reduction in retinal ischemia in an IOP rat model [6]. Elevated IOP models simulate I/R injury in glaucoma but may cause pressure-induced changes independent of ischemic damage. Few animal studies on autophagy have utilized a purely vascular I/R model without additional side-effects.

The role of autophagy in I/R injuries remains highly controversial, with some studies supporting the cytoprotective role of autophagy in retinal ganglion cells (RGCs) [2], and others demonstrating increased apoptotic cell death in the ganglion cell layer (GCL) with injection of autophagy inducers [5] or reduction in cell death with injection of autophagy inhibitors [3]. Time-dependent roles of autophagy have not been studied in the retina, but an oxygen glucose deprivation and reperfusion model in a human SH-SY5Y neuroblastoma cell line (for studying brain cells) demonstrated divergent roles of autophagy at 24 (cytotoxic) and 48 h (neuroprotective) of reperfusion [7]. A dynamic and time-dependent role of autophagic flux has also been proposed to be seen in the varying stages of cerebral brain hypoperfusion [8]. 

In this study, we investigated the (1) effect of retinal I/R injury on autophagy using both in vitro and in vivo I/R models, (2) time-dependent changes in autophagy following reperfusion in our in vivo vascular I/R model, (3) differences in autophagy expression in hyperglycemic and normoglycemic states at basal states and following I/R injury, and (4) role of autophagy (cytoprotective or cytotoxic) in retinal I/R injury. 

## 2. Results

### 2.1. Effects of Retinal I/R Injury on Electroretinogram (ERG) Responses 

Two hours of ischemia followed by two hours of reperfusion (2I2R) resulted in significant reduction in b-wave amplitude (*p* < 0.0001 for both mice groups) and increase in b-wave mean implicit time (homozygous wild-type Ins2^+/+^ mice (WT) *p* = 0.006; heterozygous Ins2^Akita/+^ mice (Akita) *p* = 0.025) in middle cerebral artery occlusion(MCAO)-injured retinae as compared with sham-treated retinae in their respective mice groups (Supplementary Figure S1). Only Akita MCAO-injured retinae demonstrated significant reduction in a-wave amplitude as compared with Akita sham-treated retinae (*p* = 0.033) (Supplementary Figure S1). The difference between the WT-treated groups were not as pronounced. MCAO-injured retinae had significantly lowered oscillatory potential (OP) 1 (WT *p* = 0.002; Akita *p* = 0.011), OP2 (WT *p* = 0.007, Akita *p* = 0.007), and OP3 amplitudes as compared with their respective sham-treated counterparts. Akita sham-treated mice demonstrated lower OP1 and 2 amplitudes as compared with their WT counterparts, but no statistically significant difference was achieved. 

**Figure 1:**
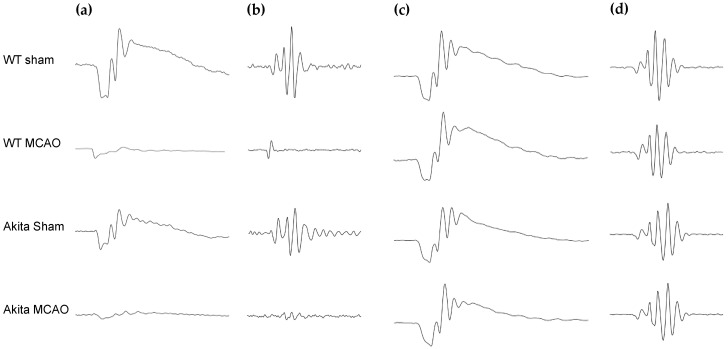
Representative waveforms of scotopic flash ERG responses in WT and Akita mice after sham or MCAO treatment for 2I2R (**a**,**b**) and 2I22R (**c**,**d**) groups at 3 cd.s/m² intensity. (**a**) a-wave and b-wave waveforms for 2I2R group; (**b**) oscillatory potential waveforms for 2I2R group; (**c**) a-wave and b-wave waveforms for 2I22R group; (**d**) oscillatory potentials waveforms for 2I22R group. Figure and caption has been adapted from a dissertation [9]. Please refer to acknowledgements section.

Contrary to the 2I2R group, there were no significant differences in a-wave, b-wave, OP amplitudes or implicit times between the two WT mice 2I22R treatment groups or between the two Akita mice 2I22R treatment groups (Supplementary Figure S2). Representative ERG waveforms are shown in Figure 1a,b (2I2R) and Figure 1c,d (2I22R). 

### 2.2. Effects of Retinal I/R Injury on Retina Morphology 

**Figure 2:**
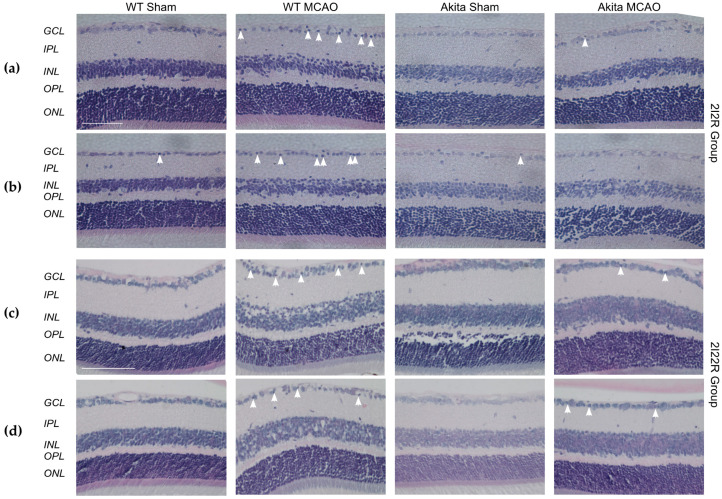
Retinal morphological samples from the 2I2R and 2I22R group in the central retina (**a**,**c**) and mid-peripheral retina (**b**,**d**). Morphological examination under 40× magnification (**a**,**b**) and 20× magnification (**c**,**d**) showed similar retinal thicknesses in all layers among all groups in both the central and mid-peripheral retinae. More pyknotic cells were observed in the WT MCAO-injured retinae (arrowheads) as compared with WT sham-treated retinae in the 2I2R group, and in the MCAO-injured retinae (arrowheads) as compared with sham-treated retinae in the 2I22R group. There is a slight increase in total retinal thickness in MCAO-injured retinae as compared to sham-treated retinae in the 2I22R group. Scale bars: 50 μm (**a**,**b**), 100 μm (**c**,**d**). Figure and caption has been adapted from a dissertation [9]. Please refer to acknowledgements section.

There was no significant difference in the respective retinal layer thicknesses (total, GCL, inner plexiform layer (IPL), inner nuclear layer (INL), outer plexiform layer (OPL) and outer nuclear layer (ONL)) between sham-operated WT and Akita mice and among sham-operated and MCAO-injured mice groups in both 2I2R (Figure 2a,b) and 2I22R (Figure 2c,d) treatment groups. 

WT MCAO-injured retinae showed a higher ratio of cells with pyknotic nuclei to number of RGCs in both the central and mid-peripheral retina as compared with WT sham-treated retinae in the 2I2R group (Supplementary Figure S3). However, no statistical significance was achieved due to the large variation between samples. Only an observed higher ratio with no statistical significance was seen in Akita sham-treated retinae as compared with their WT counterparts. There was no statistically significant reduction in the number of viable RGCs in the GCL in MCAO-injured retinae as compared with sham-treated retinae after two hours of reperfusion. 

Similarly, MCAO-injured retinae in the 2I22R group had a higher ratio of cells with pyknotic nuclei to number of RGCs in both the central and mid-peripheral retinae as compared with their respective sham-treated groups (Supplementary Figure S4). Statistical significance was seen in the central retina between the two Akita treatment groups (*p* = 0.015). A significantly lower number of viable RGCs was seen in WT MCAO-injured mid-peripheral retinae (*p* = 0.007) as compared with WT sham-treated retinae. Only an observed reduction between MCAO-injured and sham-treated groups were seen in the central retina. 

### 2.3. Effects of Retinal I/R Injury on Autophagy Marker Expression 

**Figure 3:**
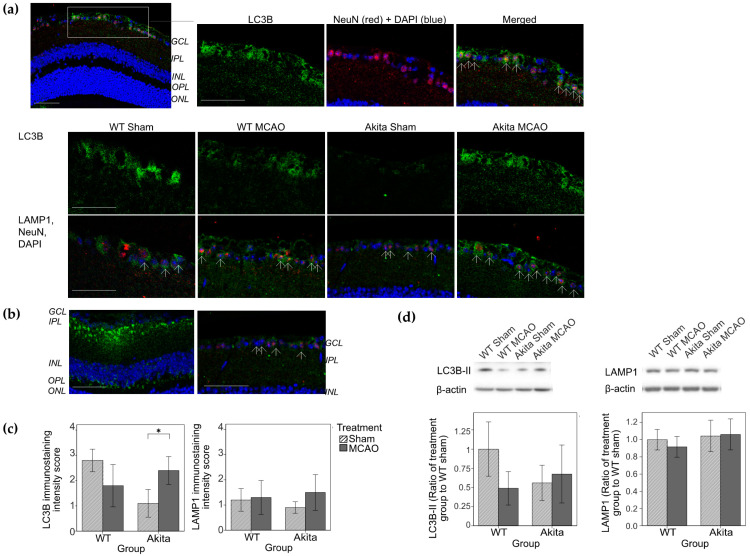
Immunohistochemical staining of PKCα, LC3B, LAMP1, and NeuN, immunohistochemical staining grading of LC3B and LAMP1 and Western blot analysis of LC3B-II and LAMP1 in the 2I2R group. (**a**) Immunohistochemical staining of LC3B (top and middle row) and LAMP1 (bottom row). Double immunolabelling of LC3 and NeuN (neuronal marker) showed colocalization of the two markers (arrows) in the GCL of the Akita MCAO-injured retina sample. LC3 immunoreactivity was increased in the GCL of Akita MCAO-injured retinae as compared with Akita sham-treated retinae. Colocalization of LAMP1 (green) and NeuN (red) (shown with DAPI) in the GCL layer (arrows). Staining of LC3 and LAMP1 was performed on adjacent slides. Scale bars: 50 μm; (**b**) Immunohistochemical staining of PKCα (left, green, shown with DAPI), LAMP1 and NeuN (right) in Akita sham-treated retinae samples to show the localization of rod bipolar cells. Colocalization of LAMP1 (green) and NeuN (red) (shown with DAPI) in the GCL layer (arrows). LAMP1 was not stained in areas of rod bipolar cells. Staining of PKCα and LAMP1 was performed on adjacent slides. Scale bars: 50 μm; (**c**) Immunohistochemical staining grading of LC3B (**left**) and LAMP1 (**right**). Score of 1 represents the weakest immunoreactivity while a score of 4 represented the highest immunoreactivity. * *p* < 0.05 as compared with Akita sham group. *n* = 5 in each group. Values represent mean with error bars +/− SD. (**d**) Western blot analysis of LC3B-II (**left**) and LAMP1 (**right**). Levels of LC3B-II were measured by Western blot analysis and normalized by β-actin. Densitometry analysis showed increased LC3B-II levels in Akita MCAO-injured retinae compared with Akita sham-treated retinae, but there was no statistically significant difference. Densitometry analysis showed no differences in LAMP1 among the four groups. *n* = 5 in each group. Values represent mean with error bars +/− SD. Figure and caption has been adapted from a dissertation [9]. Please refer to acknowledgements section.

On IHC, 2I2R resulted in upregulation of LC3B in the GCL in Akita MCAO-injured retinae as compared with Akita sham-treated retinae (*p* < 0.05 IHC score, Figure 3b). Only an observed increase in lysosomal associated membrane protein 1 (LAMP1) staining was seen but no statistical significance was detected. Co-immunolabeling of LC3B and LAMP1 with NeuN confirmed that autophagy occurred most likely in the RGCs in the GCL (Figure 3a). The location of LC3B and LAMP1 staining did not correspond with that of rod bipolar cells that were stained with PKCα (Figure 3b). 

**Figure 4:**
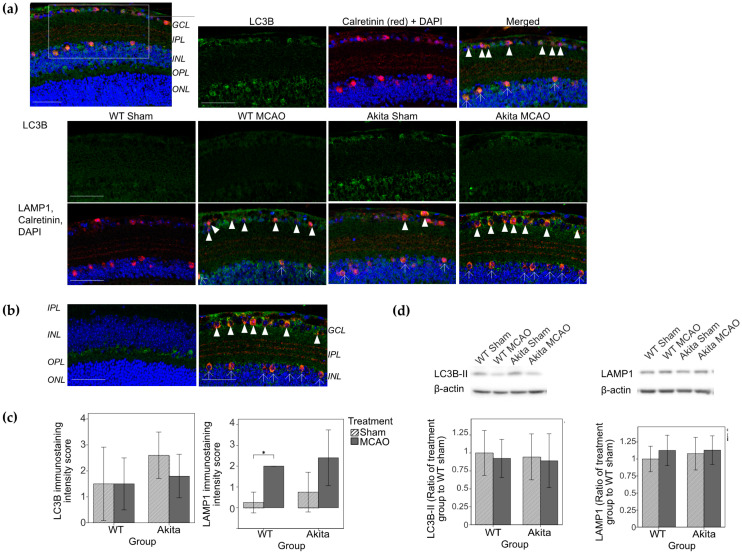
Immunohistochemical staining of calbindin, LC3B, LAMP1, and calretinin, immunohistochemical staining grading of LC3B and LAMP1 and Western blot analysis of LC3B-II and LAMP1 in the 2I22R group. (**a**) Immunohistochemical staining of LC3B (top and middle row) and LAMP1 (bottom row). Double immunolabelling of LC3 and calretinin (amacrine cell marker) showed colocalization of the two markers (arrows) in the GCL and INL of the Akita sham-treated retina sample. Colocalization of LAMP1 (green) and calretinin (red) (shown with DAPI) in the GCL layer (arrowheads) and INL (arrows). LAMP1 immunoreactivity was increased in the GCL and INL of MCAO-injured retinae as compared with sham-treated retinae. Staining of LC3 and LAMP1 was performed on adjacent slides. Scale bars: 50 μm; (**b**) Immunohistochemical staining of calbindin (left, green, shown with DAPI), LAMP1 and calretinin (right) in Akita MCAO-injured retinae samples to show the localization of horizontal cells. Colocalization of LAMP1 (green) and calretinin (red) (shown with DAPI) in the GCL (arrowheads) and INL (arrows) (same image as shown in Figure 4 (**a**) bottom right corner). LAMP1 was not stained in areas of the horizontal cells. Staining of calbindin and LAMP1 was performed on adjacent slides. Scale bars: 50 μm; (**c**) Immunohistochemical staining grading of LC3B (left) and LAMP1 (right). Score of 1 represents the weakest immunoreactivity while a score of 4 represented the highest immunoreactivity. * *p* < 0.05 as compared with WT sham group. *n* = 5 in each group. Values represent mean with error bars +/− SD.; (**d**) Western blot analysis of LC3B-II (left) and LAMP1 (right). Levels of LC3B-II were measured by Western blot analysis and normalized by β-actin. Densitometry analysis showed similar LC3B-II levels in MCAO-injured retinae as compared with their respective sham-treated counterparts. Densitometry analysis did not show significant differences in LAMP1 among the four groups. *n* = 5 in each group. Values represent mean with error bars +/− SD. Figure and caption has been adapted from a dissertation [9]. Please refer to acknowledgements section.

Contrastingly, with 22 h of reperfusion, there was no significant upregulation of LC3 but significant upregulation of LAMP1 was observed in the GCL and INL of WT MCAO-injured retinae as compared with their sham-treated counterparts (*p* = 0.025, Figure 4c). Co-immunolabelling of LAMP1 with calretinin confirmed that autophagy occurred most likely in the RGCs and amacrine cells (Figure 4a). The location of LC3B and LAMP1 staining did not correspond with that of horizontal cells that were stained with calbindin (Figure 4b). 

No statistically significant differences in autophagy marker expression were observed on Western blotting in both 2I2R and 2I22R groups (Figure 4d). 

### 2.4. Effects of Hypoxia and Hyperglycemia on Autophagy in R28 Cells 

At basal conditions, there was an observed 10% decrease in cell viability in cells induced with hyperglycemia alone (35G group) as compared with the UT-5G group. No significant decrease in cell viability was seen in the osmotic control (35M group). 

**Figure 5:**
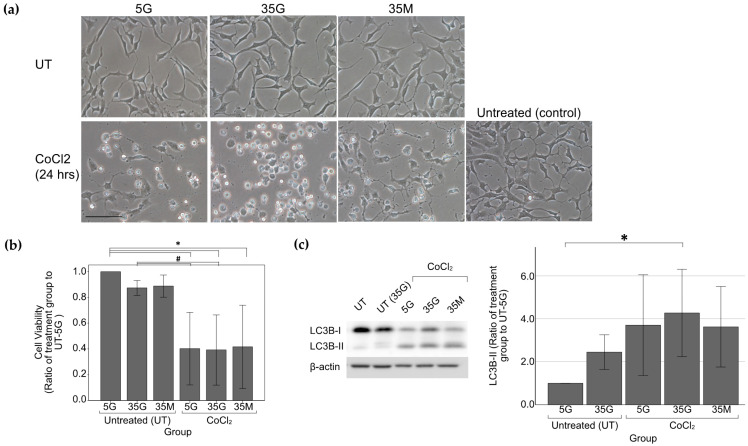
Cell morphology, cell viability and western blot analysis of LC3B-II in R28 cells under glucose treatment and/or 500 μM CoCl_2_ chemically-induced hypoxia. (**a**) Cell morphology of cells after glucose treatment alone and after 500 μM CoCl_2_ treatment. Similar cell morphology was observed among R28 cells subjected to different levels of glucose treatment and in the osmotic control. High glucose-treated cells appeared to be more susceptible to hypoxia damage as seen by the increase in cytoplasmic vacuoles. Scale bars: 100 μm; (**b**) Cell viability of cells after glucose treatment and chemically-induced hypoxia. Both low glucose and high glucose-treated cells had significantly lower cell viability than their respective untreated groups. *n* = 7 except UT-35M *n* = 5. * *p* < 0.05 as compared with UT-5G, # *p* < 0.05 as compared with UT-35G. Values represent mean with error bars +/− SD; (**c**) Western blot analysis of LC3B-II. Levels of autophagy markers LC3B-II were measured by Western blot analysis and normalized by β-actin. Densitometry analysis showed a statistically significant increase in LC3B-II levels in high glucose-treated cells subjected to hypoxia as compared with untreated cells. Glucose treatment alone (UT-35G) did not induce statistically significant LC3B-II upregulation. * *p* < 0.05 as compared with UT-5G. *n* = 7 except for UT-35G and 35M groups (*n* = 4). Values represent mean with error bars +/− SD. Figure and caption has been adapted from a dissertation [9]. Please refer to acknowledgements section.

CoCl_2_-induced hypoxia (500 μM) resulted in statistically significant decrease in cell viability and morphological changes (Figure 5a), which appeared to be more prominent in the 35G cell group. No differences were seen morphologically between the 5G and 35M group. Significant upregulation of LC3B-II levels was seen in CoCl_2_-treated cells which was most notable in the 35G group (*p* = 0.039) (Figure 5c). Only an observed 1.5-fold increase in LC3B-II levels without reaching statistical significance on statistical analysis was seen in the UT-35G group as compared with the UT-5G group.

### 2.5. Assessment of Autophagic Flux and Effects of Autophagy Inhibition under Hypoxic Conditions on R28 Cell Viability and Morphology 

To determine whether the observed increase in autophagic activity resulted from a genuine upregulation of autophagy, cells treated with chemically-induced hypoxia were incubated with an autophagy inhibitor. The 35M group was omitted from autophagy inhibition experiments in view that similar trends were observed in the 5G and 35M group (Section 2.4), suggesting that differences seen between the 5G and 35G groups were likely due to glucose level differences as opposed to osmolarity differences.

**Figure 6:**
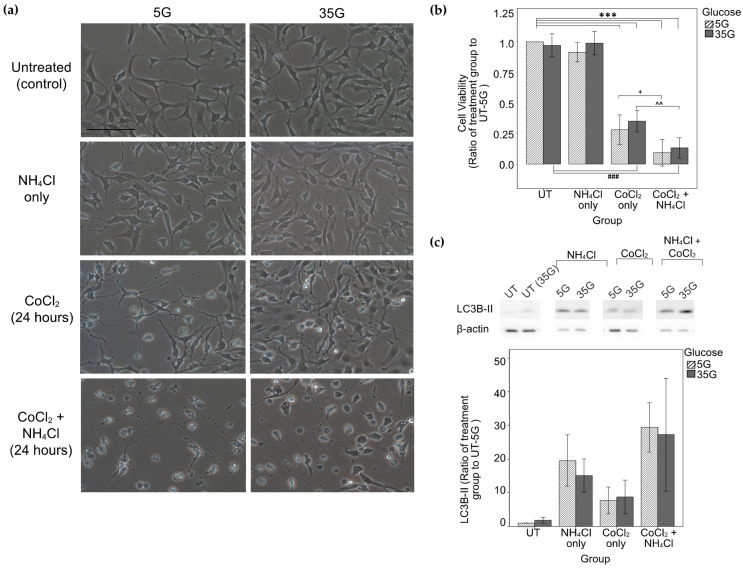
Cell morphology, cell viability and western blot analysis of LC3B-II in R28 cells after glucose treatment, NH_4_Cl treatment, 250 μM CoCl_2_ chemically-induced hypoxia or chemically-induced hypoxia with NH_4_Cl. (**a**) Cell morphology of cells subjected to CoCl_2_-induced hypoxia and autophagy inhibition. More cytoplasmic vacuoles were observed in cells treated with the autophagy inhibitor in addition to the hypoxic insult, as compared with cells treated with hypoxia only. Scale bars = 100 μm; (**b**) Cell viability of R28 cells under different treatment conditions. Both low glucose and high glucose-treated cells had significantly lower cell viability when induced with hypoxia as compared to their respective untreated groups. Those treated only with autophagy inhibitors had similar viability to their respective untreated groups. Cells treated with hypoxic insult and autophagy inhibition had lower cell viability than those treated only with hypoxic insult. *n* = 6. *** *p* < 0.001 as compared with UT-5G; ### *p* < 0.001 as compared with UT-35G; + *p* < 0.05 as compared with CoCl_2_ only-5G; ^^ *p* < 0.01 as compared with CoCl_2_ only-35G. Values represent mean with error bars +/− SD; (**c**) Levels of LC3B-II were measured by Western blot analysis and normalized by β-actin. Densitometry analysis showed an increase in LC3B-II levels in cells treated with CoCl_2_ as compared with the untreated group. Addition of NH_4_Cl to CoCl_2_ in the 5G group further increased the LC3B-II levels as compared with the CoCl_2_ only 5G group (*p* = 0.051). Figure and caption has been adapted from a dissertation [9]. Please refer to acknowledgements section.

Densitometry analysis of LC3B-II WB levels showed increase in levels in the NH_4_Cl-only groups and a six- to seven-fold increase in the CoCl_2_-only groups as compared to the UT-5G group. Significant further increases in LC3B-II levels were seen with the addition of NH_4_Cl to CoCl_2_ in the 5G group (*p* = 0.051) as compared to the CoCl_2_-only 5G group (Figure 6c). 

Treatment with 250 μM CoCl_2_ alone caused a near 60% and 55% reduction in cell viability in the 5G (*p* < 0.0001) and 35G (*p* < 0.0001) groups respectively. Addition of autophagy inhibition with NH_4_Cl to CoCl_2_-treated cells resulted in a further 50% drop in cell viability in both 5G (*p* = 0.025, compared with CoCl_2_-only 5G group) and 35G groups (*p* = 0.004, compared with CoCl2-only 35G group) (Figure 6b). This corresponded with the increased number of cytoplasmic vacuoles and alterations in cell morphology observed in chemically-induced hypoxic cells treated with autophagic inhibition (Figure 6a). NH_4_Cl treatment alone did not result in reduced cell viability (*p* = 1) or altered cell morphology (Figure 6a,b). 

## 3. Discussion

Autophagy plays a key role in maintaining cellular homeostasis. Its upregulation in response to various stimuli has been shown to mediate cytoprotective effects. We demonstrated an upregulation of autophagy after two hours of ischemia and two hours of reperfusion in the inner retinae that tapered down following 22 h of reperfusion in our in vivo experiments. 

After two hours of reperfusion, there was increased LC3B and LAMP1 staining in Akita MCAO-injured retinae as compared with their sham-treated counterparts. This demonstrated the activation of autophagy in the inner retinal layers in the RGCs (colocalization of LC3B with NeuN) and amacrine cells (colocalization of LAMP1 with calretinin). Localization of LC3B and LAMP1 immunostaining in the same areas in the GCL and INL on adjacent retinal sections suggested activity occurrence in the same cell and the formation of autolysosomes from autophagosomes and lysosomes. This finding alone, however, does not distinguish between a genuine increase in autophagic flux and the suppression of downstream processes [10,11]. 

On the contrary, the reasoning behind the observed higher basal LC3B levels in WT sham-treated mice as compared to their MCAO-injured counterparts remains unclear. It is possible that Akita MCAO-injured retinae sustained a longer and more pronounced period of autophagic upregulation due to its pre-existing higher levels of chronic oxidative stress from its chronic diabetic state prior to the retinal I/R episode. As LC3 is degraded in the autolysosomes [12], autophagic activation in WT MCAO-injured retinae may have occurred earlier than that of Akita MCAO-injured retinae, resulting in the observed reduction in LC3B staining at this particular cross section in time. 

After 22 h of reperfusion, LC3B staining in MCAO-injured retinae returned to basal levels while LAMP1 levels were increased in both WT and Akita MCAO-injured retinae. LAMP1 is a lysosomal marker that stains autolysosomes and products of the autophagic lysosome reformation process [13]. In view that LAMP1 can be considered as a marker for late stage autophagic structures [13], increased LAMP1 levels and reduced LC3B staining after 22 h of reperfusion suggested that the observed LC3B upregulation earlier represented a genuine increase in autophagic flux. This autophagic turnover possibly tapered down with prolonged reperfusion, resulting in reduced LC3 staining in the 2I22R group. 

Similar patterns of autophagy upregulation exhaustion following 24 h of reperfusion have been demonstrated in rat IOP I/R models [2] although the exact duration of autophagic activation remains to be determined. Others have demonstrated the activation of autophagosomes in RGCs for up to seven days following I/R injury [4]. Our study did not detect significant differences in autophagic markers on Western blotting of protein lysates of mice retinae from both time-points. It can be postulated that the protein lysate represented pooled data from the whole retina including areas without autophagic activation. As a result, Akita MCAO-injured retinae only demonstrated mildly elevated LC3B-II levels that was of no statistical significance as compared with their sham-treated counterparts. 

The location of autophagic activation corresponded to the primary site of injury in our retinal I/R model with the observed ERG changes. Similar results of b-wave amplitude reductions accompanied by non-apoptotic cell death in the GCL and INL have been reported in SV129EV mice [14]. A reduction in RGC count and increase in ratio of pyknotic nuclei to RGCs occurred with 22 h of reperfusion, which may imply an association between prolonged periods of reperfusion and RGC death. It remains unclear, however, as to whether cell death resulted from the initial ischemic injury and was more pronounced with prolonged reperfusion, or whether the tapering of the autophagic response triggered increased RGC loss. It has been suggested in rat models that the duration of ischemia determines the extent of b-wave recovery [15]. Our model of two hours of ischemia showed a remarkable degree of b-wave function recovery with the mean b-wave amplitudes approaching near baseline levels after 22 h of reperfusion. 

In line with the autophagic marker upregulation in the inner retinae circuits, there was significant reduction of OP amplitudes. Amplitudes returned to baseline levels after 22 h of reperfusion. OP amplitudes reflect the connections between amacrine and bipolar cells or between amacrine and ganglion cells [16]. In view of the OP amplitude recovery with prolonged reperfusion, it is possible that the decrease in autophagic response reflected the recovery from I/R injury when autophagic upregulation was no longer necessary (on the assumption that autophagy was upregulated as a cytoprotective response). 

On the other hand, a-wave amplitudes, which reflect photoreceptor function, were significantly reduced in the Akita MCAO-injured group but recovered to baseline levels by 22 h of reperfusion. This is consistent with findings of no significant a-wave changes in WT mice following 22 h of reperfusion using the same MCAO model [17], and in reports of rats that demonstrated complete recovery of a-wave amplitudes with six hours of reperfusion [15]. The mechanism behind the “regionalized sensitivity to ischemia” where the outer retinae is less sensitive to ischemia remains unclear [18], though this may explain the more rapid reversibility of a-wave changes observed. Functional ERG recovery in a- and b-wave amplitudes after 22 h of reperfusion were similar between WT and Akita MCAO-injured mice and did not suggest that Akita mice had poorer functional outcomes and ERG recovery with I/R injury. 

Following the findings in our in vivo model, we tested the role of autophagy using a R28 cell model. In hyperglycemic culture conditions alone, there was no statistically significant reduction in cell viability in R28 cells. Existing reports have demonstrated variable effects of hyperglycemia in primary rat retinal neural cells and mixed primary retinal cultures [19,20,21]. The discrepancy in results may be partly attributed to the heterogenous nature of R28 cells and differences in duration of glucose incubation. Despite differentiation of R28 cells into a more neuronal phenotype using pCPT-cyclic AMP [22,23], glial and photoreceptor components may remain. Indeed, one study suggested that Müller glia may protect retinal neuronal cells against hyperglycemia-induced toxicity [21]. 

Our study also observed non-statistically significant higher levels of autophagy marker expression (LC3B-II) in hyperglycemic cells as compared to normoglycemic cells at basal culture conditions. Lopes d Faria et al. previously described correlations between high glucose levels and increased autophagic flux dysfunction and apoptosis in rMC-1 cells [24]. Overactivation of autophagy may be implicated in cytotoxicity. Alternately, it could be a homeostatic cytoprotective upregulation for elimination of ROS in diabetic states where oxidative stress, production of ROS and misfolded proteins is increased [25]. While the rate of LC3-II (active phagosome membrane-bound form) increase (as opposed to measurements of its cytosolic form, LC3-I) is speculated to correspond with the number of autophagosomes [10,12], upregulation of LC3-II alone may result from autophagic activation or inactivation of downstream components of the autophagic pathway [10,26]. The pattern of conversion from LC3-I to II is also cell-type dependent and affected by the nature of stress that has been inflicted in cells [26]. 

With chemically-induced hypoxia using CoCl_2_, our study confirmed a decrease in cell viability and upregulation of LC3B-II with CoCl_2_-induced hypoxia. This corresponds to other reported studies of similar nature using other retinal culture models [27,28,29]. Hypoxia-induced apoptosis with 500 μM of CoCl_2_ has been confirmed in previous studies of R28 cells by the stabilization of HIF-1α and caspase3 [30]. Cells cultured under hyperglycemic conditions were more prone to CoCl_2_-induced hypoxic insult on morphological examination, with an observed decrease in cell viability as compared to normoglycemic cells. No objective differences in cell viability were detected upon MTS examination. No differences were observed in the mannitol osmotic control group and control group, indicating that effects observed in the 35G group were not due to osmolality differences. Indeed, there have been discrepancies in previous reports regarding effects of high glucose on retinal cell survival. One study reported no effects of hyperglycemia on R28 cell survival [20] while others have reported RGC apoptosis in animal models of diabetic retinopathy [14,31,32], and neurotoxicity and cell death in primary rat retinal neural cells under high glucose conditions [33,34,35]. The lack of statistically significant results observed in our studies may partly be explained by differences in glucose incubation duration as compared with other reports and the heterogenous nature of R28 cells. 

Regarding the nature of our observed upregulation of LC3B-II under hypoxic conditions, further in vitro LC3 turnover assays confirmed that LC3B-II accumulation was significantly increased in the presence of a lysosomal degradation inhibitor (NH_4_Cl) that blocks the downstream autophagic pathway [10,12] as compared with its absence in chemically-induced hypoxia (*p* = 0.051 for 5G group). This is indicative of a true increase in autophagic flux [10,26,36]. A similar increase was seen in the 35G group, although no statistical significance was achieved. It has been postulated that there are higher basal rates of autophagic flux in hyperglycemic states [10], while other studies of rMCs have demonstrated defective and compromised autophagic flux responses in hyperglycemic states [24]. In conditions with high basal levels of autophagic flux, further additional changes in LC3 turnover may be difficult to be detected with LC3 turnover assays [10]. The above factors, along with variations in responses between groups of cells, may contribute to the observed phenomenon. 

Autophagy inhibition with CoCl_2_-induced hypoxia resulted in significantly decreased in R28 cell viability, an increase in cytoplasmic vacuoles and increased alterations in cell morphology as compared with CoCl_2_-induced hypoxia alone. Our in vitro results using retinal precursor R28 cells that have been differentiated into a more neuronal phenotype correlate well with the increased cell death and autophagic activation seen in the inner retinae in our in vivo model. However, R28 cells may remain to have both glial and photoreceptor cell phenotypes after differentiation using pCPT-cyclic AMP given its heterogenous nature, and the quantification and expression of specific proteins for neuronal, glial, or photoreceptor components was not performed. Such detrimental effects of autophagy inhibition in hypoxic injuries were demonstrated at one specific time point. However, further time-point studies would be needed to characterize potential time-dependent changes in autophagic markers and effects of autophagic inhibition in hypoxia given the dynamic nature of autophagy. 

## 4. Materials and Methods

### 4.1. In Vivo Study Design 

All animal studies were approved by the Committee on the Use of Live Animals in Teaching and Research in The University of Hong Kong and conducted in accordance with the Cap. 340 Animals (Control of Experiments) Ordinance in Hong Kong. Wild-type female C57BL/6J from the Jackson Laboratory were inbred with heterozygous male C57BL/6-Ins2^Akita/^J mice (Ins2^Akita/+^). Heterozygous Ins2^Akita/+^ (Akita) and homozygous wild-type Ins2^+/+^ (WT) male offspring aged 11–15 weeks were selected. Polymerase chain reaction for insulin 2 gene amplification was performed to confirm the genotyping of mice (Supplementary Figure S5). 

WT and Akita mice were assigned to one of four experimental groups: (1) retinal I/R injury with two hours of reperfusion (middle cerebral artery occlusion MCAO 2I2R); (2) sham operation with two hours of reperfusion (sham 2I2R); (3) retinal I/R injury with 22 h of reperfusion (MCAO 2I22R); and (4) sham operation with 22 h of reperfusion (sham 2I22R). Retinal I/R injury was performed using an intraluminal right-sided MCAO model as described previously [37,38,39], where the intra-luminal filament inserted into the middle cerebral artery to induce ischemia was removed after two hours to allow for 2 or 22 h of reperfusion. This model results in effective occlusion of the blood supply to the ipsilateral retina as the ophthalmic artery is located closely to the MCA in mice. Neurological scoring and ERG were performed before animals were euthanized by ketamine and xylazine. Right eyecups of mice from each experimental group were extracted for retinal morphological examination or protein extraction for Western blot (WB) analysis. 

#### 4.1.1. ERG

All mice were dark adapted for four hours (2I2R group) or >12 h (2I22R group) and anesthetized intraperitoneally with a mixture of ketamine and xylazine. Pupils were anesthetized topically and dilated with 0.5% alcaine and 1% Mydricyl, respectively. Signals were measured using a ground electrode in the forehead, reference needle in the tail, and gold electrodes on the cornea. 15 3 cd.s/m^2^ white flashes each separated by five second intervals, followed by five minutes of dark adaptation and 15 10 cd.s/m^2^ white flashes with five second intervals in between each flash, were delivered using the ColorDome Ganzfeld System (Diagnosys, Lowell, MA, USA). Signals were selected, analyzed and amplified by the Epsion V5 Software (Diagnosys). Measurements of the amplitude and implicit times of a-waves, b-waves, and oscillatory potentials were taken. 

#### 4.1.2. Tissue Preparation 

Eyecups for morphological examination were fixed in 4% paraformaldehyde (PFA) in phosphate-buffered saline and dehydrated with a graded ethanol series and chloroform at room temperature prior to paraffin embedding. Shandon Histocentre2 Embedding Station, Midwest was used for paraffin embedding. 5 μm-thick sagittal sections were sliced parallel to the optic nerve using a microtome (HM 315 Microtome, Microm) and mounted onto microscope slides. Sections with the optic nerve stump were selected for histological examination and immunohistochemical staining. 

#### 4.1.3. Hematoxylin and Eosin (H&E) and Immunohistochemical Staining 

Deparaffinized retinal sections were stained with H&E staining. H&E-stained retinal sections were photographed using an upright microscope (Eclipse 80i, Nikon, Tokyo, Japan) at two areas (measuring 225 × 300 μm each) from the central retina (100 μm from the optic stump) and two areas at the mid-peripheral retina (100 μm from the end of the peripheral retina) for counting of viable RGCs and cells with pyknotic nuclei in the GCL and measurements of the retinal layer thicknesses in each retinal section. The average of the two measurements was taken for statistical analysis. *n* = 5 for each group.

For immunohistochemistry (IHC), antigen retrieval was performed by incubating sections using proteinase K (1:500 proteinase K:PBS) for four minutes. 10% normal goat serum (#G9023, Sigma-Aldrich, St. Louis, MO, USA) or 10% donkey serum (#D9663, Sigma) in primary diluent was used to block non-specific binding. Sections were incubated with anti-LC3B (NB100-2220; for autophagy), anti-LAMP1 (ab24170, Abcam, Cambridge, UK; for autophagy), anti-calbindin D-28K (AB1778, Millipore, Burlington, MA, USA; for horizontal cells), anti-calretinin (Sc-11644, Santa Cruz Biotechnology, Dallas, TX, USA; for amacrine cells), anti-PKCα (Sc-208, Santa Cruz Biotechnology; for rod bipolar cells), or anti-neuronal nuclei (MAB377, Millipore; for RGCs) antibodies at 4 °C overnight. Sections were further incubated with corresponding 488 donkey anti-rabbit (A21206, Invitrogen, Waltham, MA, USA) or 568 donkey anti-goat secondary antibodies (A11057, Life Technologies, Carlsbad, CA, USA) for one hour for signal visualization. 4,6-diamidino-2- phenylindole (DAPI) (1:1000) was used for cell nuclei staining. To visualize the localization of autophagy markers in the GCL and INL, double immunolabeling was performed for autophagy markers with NeuN or calretinin respectively. Adjacent slides were used for LC3B and LAMP1 staining to evaluate for colocalization of markers. Images were captured using a confocal microscope (Zeiss LSM 700, Zeiss, Jena, Germany) and the intensity of IHC staining was graded in a double-blinded fashion on a scale from one to four [38,39,40,41,42,43], with one being the least staining. IHC experiments were performed on all samples at the same time. *n* = 5 in each group.

#### 4.1.4. WB Analysis 

For protein extraction, eyecups were snap frozen using liquid nitrogen and stored in −80 °C prior to further processing. Supernatant was prepared by lysing retina protein samples with RIPA lysis buffer (0.15 M NaCl, 5 mM EDTA (pH 8.0)), protease inhibitor (Roche, Basel, Switzerland) and phosphatase inhibitor (Calbiochem, San Diego, CA, USA), followed by vortexing and centrifugation. Protein concentrations were measured using the Quick Start^TM^Bradford Protein Assay (Bio-Rad, Hercules, CA, USA). The mixture of supernatant with 6× loading buffer was denatured at 98 °C. 10 or 20 μg of samples were separated using 10% or 15% gels and electroblotted onto a polyvinylidene fluoride (PVDF) membrane. Membranes were incubated with mouse anti-actin clone c4 (MAB1501, Millipore), rabbit anti-LC3B (#2775, Cell Signaling Technology, Danvers, MA, USA) or rabbit anti-LAMP1 (ab24170, Abcam) primary antibodies at 4 °C overnight on a shaker. This was followed by further incubation with the corresponding horseradish peroxidase-labeled secondary antibodies (anti-rabbit or anti-mouse, BD Biosciences, Franklin Lakes, NJ, USA) for one hour. Amersham^TM^ ECL^TM^ or ECL^TM^ Prime Western Blotting Detection Reagent (GE Healthcare, Chicago, IL, USA) and myECLTM Imager (Thermo Fisher Scientific, Waltham, MA, USA) was used for protein band detection. Protein levels were normalized using β-actin and semi-quantification of protein bands was performed using ImageJ. *n* = 5 in each group. 

### 4.2. In Vitro Study Design 

Retinal precursor cell (R28 cells, KeraFAST Inc., Boston, MA, USA) was cultured in Dulbecco’s modified Eagle’s medium (DMEM)—Low Glucose Medium (5 mM glucose) (DMEM-LG, Gibco, Waltham, MA, USA) supplemented with 1% penicillin/streptomycin (Life Technologies), 1% MEM non-essential amino acids (Gibco), 1% MEM vitamins (Gibco), 1% L-glutamine (Gibco), gentamicin (100 μL per 50 mL) (G418 sulfate, Merck, Kenilworth, NJ, USA), and 10% fetal bovine serum. Cells were passaged at 80% confluence. 

Cells were seeded at a density of 2.5 × 10^4^ cells/ well (96-well plate, IWAKI, Tokyo, Japan) or 1 × 10^5^ cells/ well (12-well plate, IWAKI) on laminin-coated (Cultrex^®^ Mouse Laminin I, Trevigen^®^, Gaithersburg, MD, USA) plates respectively for 24 h prior to treatment. There have been no standardized models for mimicking hyperglycemic states as various culture mediums and basal glucose requirements are required for different cell types. In view that glucose levels of 25–50 mM have been reported [33] with most retinal neuronal models using levels of 25–35 mM, 30 mM of additional glucose was added to mimic a hyperglycemic state (35G group), while 30 mM of mannitol was added for the osmotic control (35M group). 250 μM of pCPT-cyclic AMP (Sigma-Aldrich) was added every 24 h when cell medium was changed to differentiate cells into a more neuronal-like phenotype [22]. Chemical hypoxia was induced by incubating cells with 500 μM CoCl_2_ (CoCl_2_; Sigma-Aldrich) [17,27,44,45] 24 h after glucose treatment was added. A control group (untreated group, UT) for each respective glucose group was incorporated into the experiments. For autophagy inhibition, 20 mM of ammonium chloride NH_4_Cl, a lysosomotropic agent, and 250 μM CoCl_2_ were added to the cell culture medium at the same time. 

#### 4.2.1. Cell Morphology and Viability 

Cells were photographed at 20× magnification using a light microscope (Eclipse 80i, Nikon) prior and after each stage of treatment was added. 

Cell viability was assessed using the CellTitre 96^®^ Aqueous Non-radioactive Cell Proliferation Assay (Promega, Madison, WI, USA) as per the product protocol. After completion of experiments, cells were incubated with a mixture of 3-(4,5-dimethylthiazol-2-yl)-5-(3-carboxymethoxyphenyl)-2-(4-sulfophenyl)-2H- tetrazolium, inner salt (MTS), phenazine methosulfate (PMS), and medium. Absorbance at 490 nm was measured using a microplate reader (ELx800^TM^ Absorbance Microplate Readers; BioTek, Winooski, VT, USA). The average of the triplicate cell viability readings of each experimental group was taken for analyses after deducting the background control (MTS, PMS, medium mixture) readings. 

#### 4.2.2. WB Analysis 

Cells were trypsinized with 0.125% Trypsin-EDTA (0.25% 1× Trypsin-EDTA, Gibco, diluted to 0.125% using PBS for cell-culture). Cells were lysed as described above in Section 4.1.4 and incubated with LC3B and LAMP1 antibodies. 

#### 4.2.3. Statistical Analyses 

Statistical analyses were performed with 0.05 as the significance level. Non-parametric Kruskal–Wallis with a Dunn’s multiple comparison test was used to analyze IHC studies. Normality and homogeneity of variances were checked for all WB results, retinal thickness measurements, cell count studies, ERG and cell viability analyses before a one-way analysis of variance (ANOVA) with the appropriate corresponding post-Hoc test (Bonferroni test for equal variances assumed, or Dunnett’s T3 test for equal variances not assumed) was performed. Experimental groups for in vitro studies were compared with the untreated UT-5G group for analyses unless otherwise specified. Experimental mice groups for in vivo studies were compared with their respective sham-treated counterparts unless otherwise specified. 

## 5. Conclusions

Our in vivo studies suggested an upregulation of autophagy in retinal I/R injury in the inner retinae after two hours of ischemia and two hours of reperfusion, with a tapering of the autophagic response with 22 h of reperfusion. Autophagic activation in the GCL and INL correlated well with functional ERG changes. Injury appeared to be reversible with two hours of ischemia, as reflected by the return of a-wave, b-wave, and OP amplitudes to baseline levels following 22 h of reperfusion, correlating with the tapering of the autophagic response seen on IHC studies. The reversibility of ERG changes represents potential therapeutic timeframes for interventions. 

A true increase in autophagic flux was confirmed with in vitro studies with R28 cells where LC3-II turnover studies showed increased accumulation of LC3-II in hypoxic cells treated with lysosomotropic agent NH_4_Cl as compared with those receiving CoCl_2_-induced hypoxic treatment alone. We demonstrated worsened cell survival with alterations in cell morphology with pharmacological autophagic inhibition delivered at the same time as the 24-h CoCl_2_-induced hypoxic injury. NH_4_Cl treatment alone did not result in such structural and viability changes. These results support the cytoprotective role of autophagic upregulation in retinal I/R injuries at this particular time point, though given its dynamic nature, autophagy modulation at differing time points may account for varying functional roles. 

Current clinical treatments for retinal I/R injuries remain focused on reducing oxidative stress and apoptosis as the use of autophagy inducers and inhibitors have been limited to experimental settings. However, a growing understanding of the role of autophagic regulation and its effect on cell death in retinal I/R injuries may facilitate the development of clinical neuroprotective strategies using autophagic modulating properties of both new and existing drugs. 

## Data Availability

All relevant data is contained within the article, its supplementary materials and in “Tang, L.H.C. Autophagy in Retinal Ischemia/Reperfusion Injuries. Master’s Thesis, The University of Hong Kong, Hong Kong, 2019”.

## Supplementary Materials

The following are available online at https://www.mdpi.com/article/10.3390/ijms22168446/s1.
