# Supplementary material for: Autophagic Upregulation Is Cytoprotective in Ischemia/Reperfusion-Injured Retina and Retinal Progenitor Cells
Source: Int J Mol Sci. 2021 Aug 6;22(16):8446. doi: 10.3390/ijms22168446 (PMC8395130; doi:10.3390/ijms22168446)

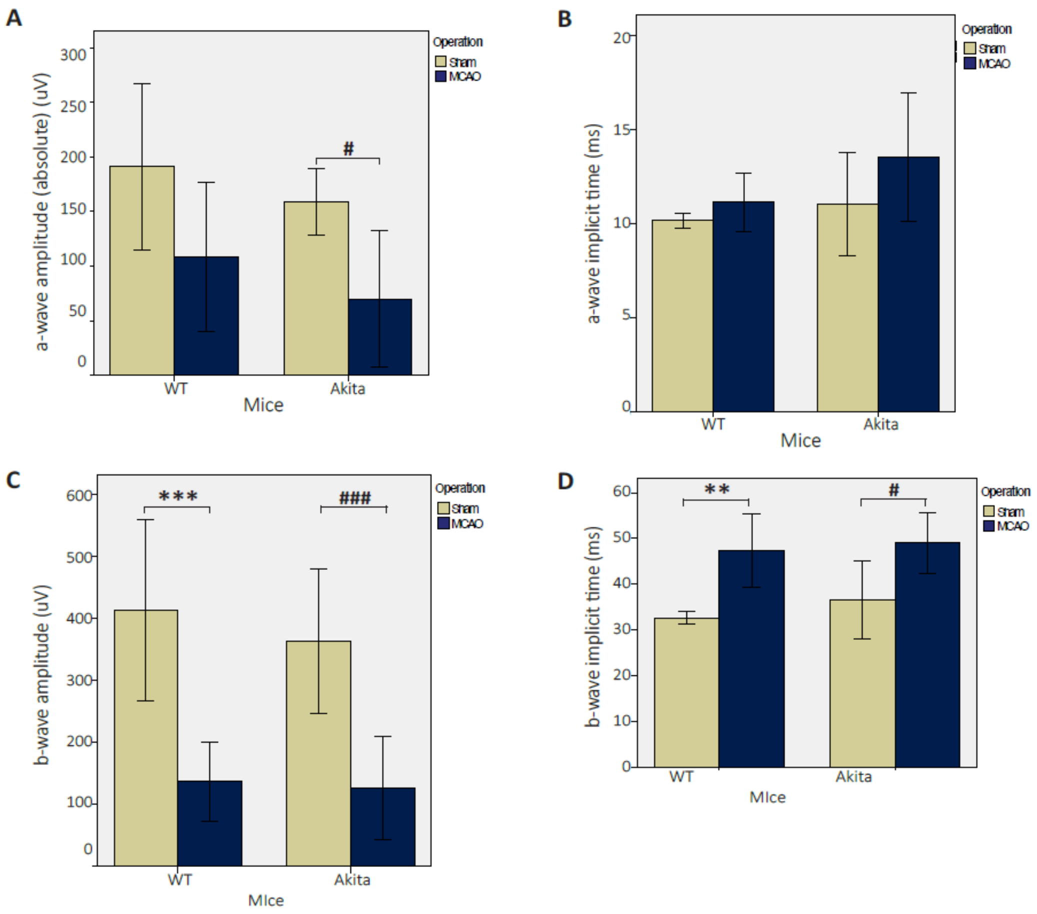

Supplementary Figure S1. Scotopic flash ERG responses of a- and b-waves in WT and Akita mice after sham or MCAO treatment (2I2R group) at 3 cd.s/m2 intensity (**A**) a-wave amplitude (**B**) a-wave implicit time (**C**) b-wave amplitude (**D**) bwave implicit time. n = 7 for WT sham group, n = 8 for WT MCAO group and Akita MCAO group, n = 9 for Akita sham group. # $p < 0.05$, ### $p < 0.001$ as compared with Akita sham. ** $p < 0.01$, *** $p < 0.001$ as compared with WT sham. Values represent mean with error bars +/- SD. (Figure and caption has been adapted from a dissertation[9]. Please refer to acknowledgements section.)

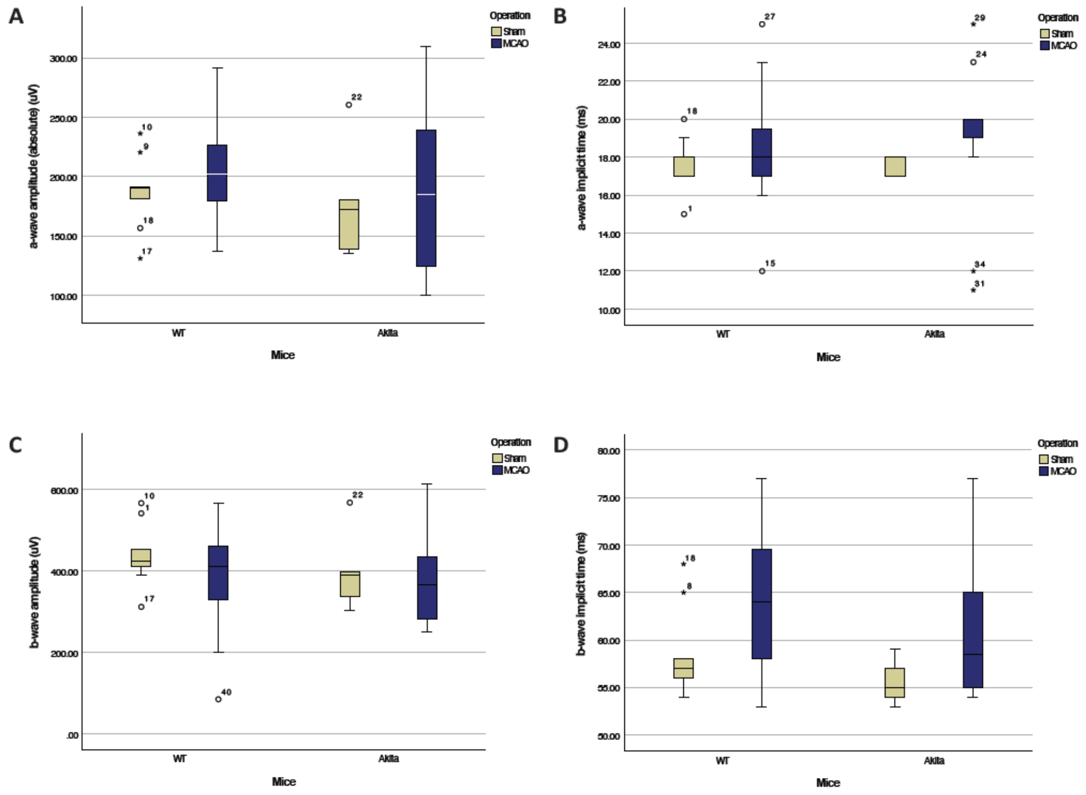

Supplementary Figure S2. Scotopic flash ERG responses of a- and b-waves in WT and Akita mice after sham or MCAO treatment (2I22 group) at 3 cd.s/m2 intensity. (**A**) a-wave amplitude (**B**) a-wave implicit time (**C**) b-wave amplitude (**D**) bwave implicit time. *n* = 9 for WT sham group. *n* = 15 for WT MCAO group. *n* = 5 for Akita sham group. *n* = 14 for Akita MCAO. (Figure and caption has been adapted from a dissertation[9]. Please refer to acknowledgements section.)

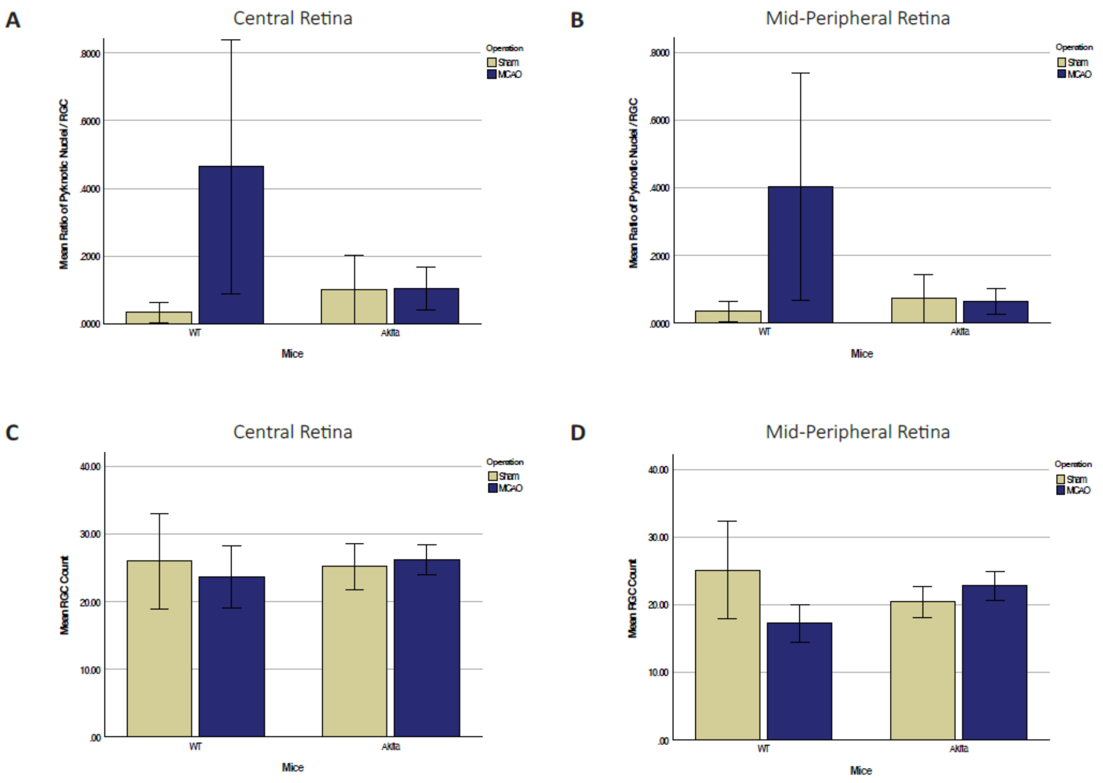

Supplementary Figure S3. RGC and pyknotic cell count in the GCL (2I2R group). (**A, B**) Ratio of cells with pyknotic nuclei to RGCs in the central (**A**) and mid-peripheral retina (**B**). (**C, D**) Number of RGCs in the GCL in the central (**C**) and midperipheral retina (**D**). No statistical significance detected. n = 5 in each group. Values represent mean with error bars +/- SD. (Figure and caption has been adapted from a dissertation[9]. Please refer to acknowledgements section.)

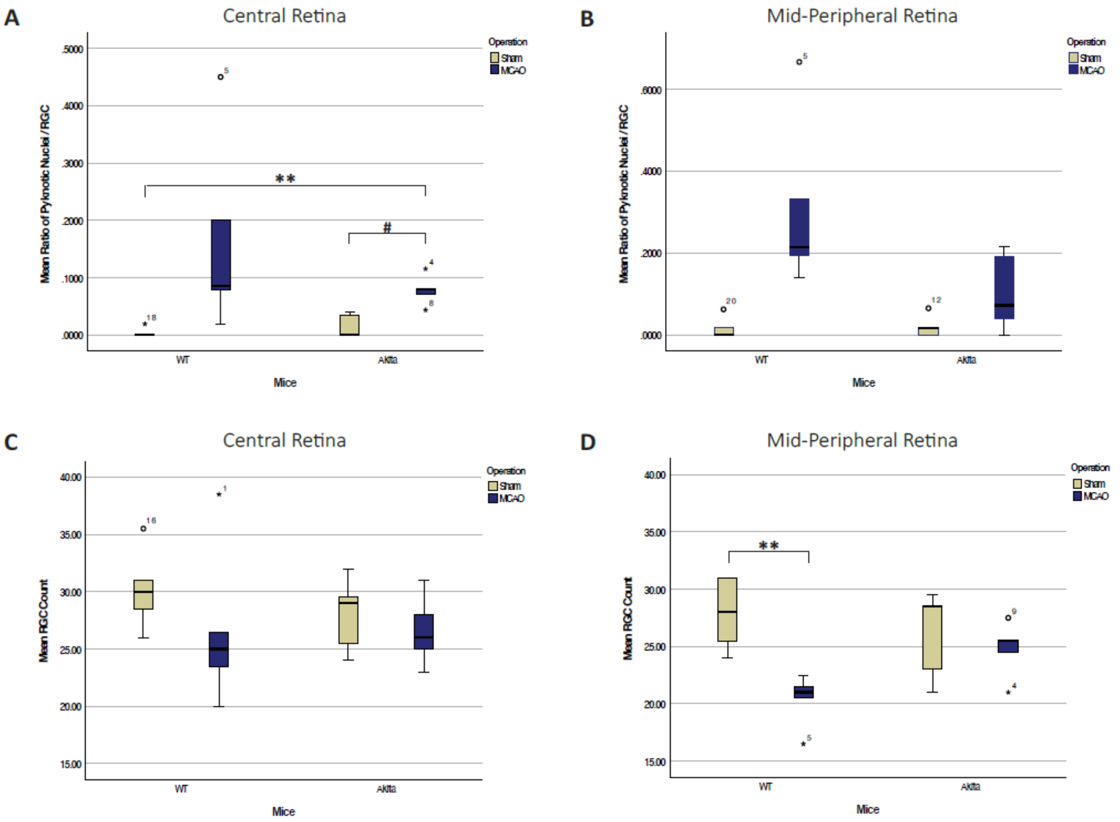

Supplementary Figure S4. RGC and pyknotic cell count in the GCL (2I22R group) (**A, B**) Ratio of cells with pyknotic nuclei to RGCs in the central (**A**) and mid-peripheral retina (**B**). (**C, D**) Number of RGCs in the GCL in the central (**C**) and midperipheral retina (**D**). The RGC cell count was slightly lower in WT MCAO-injured retinae as compared with WT shamtreated retinae in the mid-peripheral retinae, with statistical significance detected in the mid-peripheral retinae. $n = 5$ in each group. ** $p < 0.01$ as compared with WT sham group. # $p < 0.05$ as compared with Akita sham group. (Figure and caption has been adapted from a dissertation[9]. Please refer to acknowledgements section.)

Supplementary Figure S5. Examples of PCR analysis for genotyping of Akita mice

Lane	1	2	3	4	5	6
	Marker	A8/E8	A7/C2	A7/C1	A7/13-1	A8/5-3
Result	---	WT	WT	WT	Akita	WT

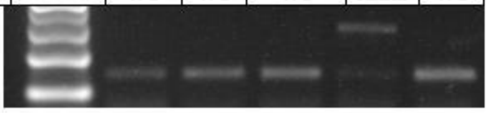

Lane	1	2	3	4	5	6	7	8	9	10
	Marker	G1-2	G1-3	A8/B1	A8/B2	A8/B3	A8/B4	A8/B5	A8/B6	A8/B7
Result	---	Akita	WT	WT	Akita	Akita	WT	WT	WT	WT

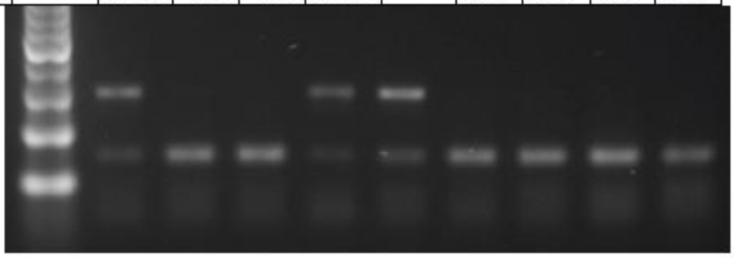

Lane	1	2	3	4	5	6	7	8	9	10	11	12	13
	Marker	A8/5-1	A8/5-2	A8/5-4	A7/D1	A7/D2	A7/D3	A2/9-1	A2/9-2	A2/9-3	A2/9-4	A2/9-5	A7/13-2
Result	---	Akita	Akita	Akita	Akita	Akita	Akita	WT	Akita	WT	WT	Akita	Akita

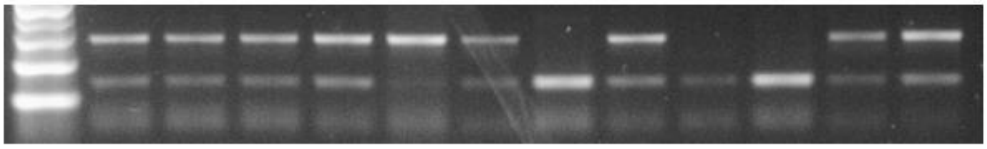

Lane	1	2	3	4	5	6	7	8	9	10	11	12	13
	Marker	/	/	A7/12-8	A7/12-9	A8/5-5	A8/5-6	A8/5-7	A7/D4	A7/D5	A7/D6	A7/D7	A7/D8
Result	---			Akita	Akita	Akita	Akita	WT	Akita	WT	Akita	Akita	Akita

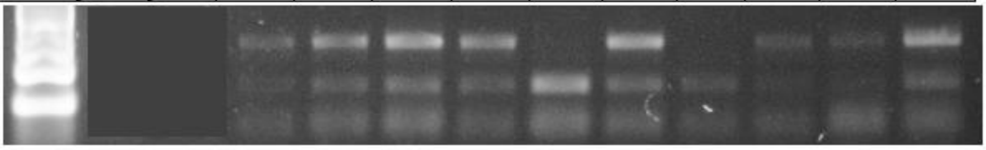

Lane	1	2	3	4	5	6	7	8
	Marker	G1/20 -1	G1/20- 1	G1/11- 1	G1/11- 2	/	G1/11- 4	G1/11 -5
**Result**	---	**WT**	**WT**	**Akita**	**Akita**		**Akita**	**Akita**

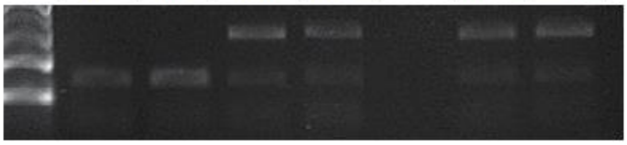

Lane	1	2	3	4	5
	Marker	F2-6	G1-1	G1-2	G1-3
**Result**	—	**Akita**	**WT**	**Akita**	**WT**

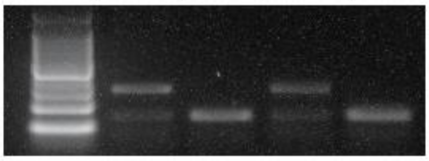

Lane	1	2	3	4	5	6	7	8	9	10	11	12	13	14	15	16
	F1-1	F1-2	F1-4	/	F2-7	F3-8	F5-15	F6-19	Marker	/	/	G1-3	G1-4	G2-1	G2-2	G2-3
**Result**	**Akita**	**Akita**	**Akita**		**Akita**	**Akita**	**Akita**	**Akita**				**WT**	**Akita**	**Akita**	**Akita**	**Akita**

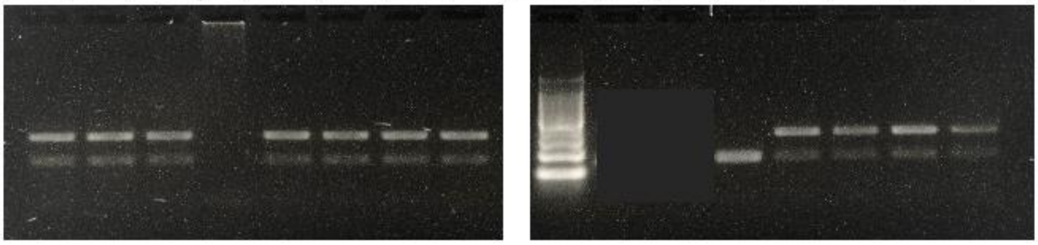

Supplement: Supplementary file 1 [file ijms-22-08446-s001.zip › ijms-1323147-supplementary.pdf]
